# Structural MRI Study of the Planum Temporale in Individuals With an At-Risk Mental State Using Labeled Cortical Distance Mapping

**DOI:** 10.3389/fpsyt.2020.593952

**Published:** 2020-11-24

**Authors:** Yoichiro Takayanagi, Sue Kulason, Daiki Sasabayashi, Tsutomu Takahashi, Naoyuki Katagiri, Atsushi Sakuma, Noriyuki Ohmuro, Masahiro Katsura, Shimako Nishiyama, Mihoko Nakamura, Mikio Kido, Atsushi Furuichi, Kyo Noguchi, Kazunori Matsumoto, Masafumi Mizuno, J. Tilak Ratnanather, Michio Suzuki

**Affiliations:** ^1^Department of Neuropsychiatry, University of Toyama Graduate School of Medicine and Pharmaceutical Sciences, Toyama, Japan; ^2^Arisawabashi Hospital, Toyama, Japan; ^3^Center for Imaging Science and Institute for Computational Medicine, Department of Biomedical Engineering, Johns Hopkins University, Baltimore, MD, United States; ^4^Research Center for Idling Brain Science, University of Toyama, Toyama, Japan; ^5^Department of Neuropsychiatry, Toho University School of Medicine, Tokyo, Japan; ^6^Department of Psychiatry, Tohoku University Hospital, Sendai, Japan; ^7^Osaki Citizen Hospital, Sendai, Japan; ^8^Health Administration Center, University of Toyama, Toyama, Japan; ^9^Department of Radiology, University of Toyama Graduate School of Medicine and Pharmaceutical Sciences, Toyama, Japan; ^10^Kokoro no Clinic OASIS, Sendai, Japan

**Keywords:** psychosis, planum temporale (PT), clinical high risk (CHR) for psychosis, labeled cortical distance mapping, schizophrenia

## Abstract

**Background:** Recent studies have demonstrated brain structural changes that predate or accompany the onset of frank psychosis, such as schizophrenia, among individuals with an at-risk mental state (ARMS). The planum temporale (PT) is a brain region involved in language processing. In schizophrenia patients, gray matter volume reduction and lack of normal asymmetry (left > right) of PT have repeatedly been reported. Some studies showed progressive gray matter reduction of PT in first-episode schizophrenia patients, and in ARMS subjects during their development of psychosis.

**Methods:** MRI scans (1.5 T field strength) were obtained from 73 ARMS subjects and 74 gender- and age-matched healthy controls at three sites (University of Toyama, Toho University and Tohoku University). Participants with ARMS were clinically monitored for at least 2 years to confirm whether they subsequently developed frank psychosis. Cortical thickness, gray matter volume, and surface area of PT were estimated using FreeSurfer-initiated labeled cortical distance mapping (FSLCDM). PT measures were compared among healthy controls, ARMS subjects who later developed overt psychosis (ARMS-P), and those who did not (ARMS-NP). In each statistical model, age, sex, intracranial volume, and scanning sites were treated as nuisance covariates.

**Results:** Of 73 ARMS subjects, 18 developed overt psychosis (12 schizophrenia and 6 other psychoses) within the follow-up period. There were no significant group differences of PT measures. In addition, significant asymmetries of PT volume and surface area (left > right) were found in all diagnostic groups. PT measures did not correlate with the neurocognitive performance of ARMS subjects.

**Discussion:** Our results suggest that the previously-reported gray matter reduction and lack of normal anatomical asymmetry of PT in schizophrenia patients may not emerge during the prodromal stage of psychosis; taken together with previous longitudinal findings, such PT structural changes may occur just before or during the onset of psychosis.

## Introduction

Structural anomaly of the superior temporal gyrus (STG) in individuals with schizophrenia has been shown in many studies (1). The planum temporale (PT), a multisensory region located on the posterior surface of STG, is involved in auditory speech processing and language comprehension (2). A recent meta-analysis revealed a robust bilateral volume reduction of PT (left: Cohen's *d* = −0.775; right: Cohen's *d* = −0.349) in schizophrenia patients compared with healthy controls (HC) (3). Although PT is thought to be an asymmetric (left > right) structure in healthy subjects (4), the leftward laterality is reduced or reversed in schizophrenia (5, 6). The reduction of leftward asymmetry of PT volume was also reported in first-degree relatives of schizophrenia (7). Some studies also demonstrated progressive gray matter reduction of PT in first-episode schizophrenia patients (8, 9) (the mean intervals between scans were 1.5 and 2.7 years, respectively).

Psychotic disorders, such as schizophrenia, are characterized by disabling features including low functional outcomes (10), prominent physical health problems (11) and premature mortality (12). Substantial attempts have therefore been made to predict, delay, or even prevent overt psychosis in individuals with an at-risk mental state (ARMS) for psychotic disorders (13, 14). We previously reported the progressive gray matter reduction of PT during the transition to psychosis in ARMS subjects (15). However, the precise chronology of PT structural changes remains unclear, i.e., whether it begins before or after the onset of psychosis.

Labeled cortical distance mapping (LCDM) is a novel image analyzing tool that computes the distances between labeled gray matter voxels and the gray/white matter cortical surface. LCDM can reliably characterize the morphometry of the laminar cortical mantle of cortical structures, such as cortical thickness and gray matter volume (16). Since this method is applied to a specific region of interest (ROI), it offers better tissue segmentation and is thus less susceptible to signal intensity inhomogeneity than whole-brain analyses. Using LCDM, we previously reported reduced thickness and less asymmetry in the volume of PT among schizophrenia patients compared with controls (17).

In the present study, we analyzed the PT morphology in ARMS subjects and HC using LCDM. We then examined whether PT structural changes had predated the future onset of frank psychosis in individuals with ARMS. In addition, we tested the associations between neurocognitive performance and PT measures among a sub-sample (*n* = 36) of ARMS subjects.

## Methods

### Participants

We recruited 73 ARMS subjects and 74 HCs at three sites (Toho University Hospital, Tohoku University Hospital and Toyama University Hospital) having specialized clinical services for ARMS (18). The ARMS phase is defined by having either attenuated psychotic symptoms, brief limited intermittent psychotic symptoms or genetic risk and deterioration in functioning (19). We used the Comprehensive Assessment of At-Risk Mental State (CAARMS) (20) (University of Toyama and Tohoku University) or the Structured Interview for Prodromal Syndrome/Scale of Prodromal Symptoms (SIPS/SOPS) (21) (Toho University) for the diagnosis of ARMS. ARMS subjects were clinically followed for at least 2 years after MRI scanning to confirm whether they developed full-blown psychosis (ARMS-P) or not (ARMS-NP). The mean follow-up period for the ARMS-NP subjects was 6.5 ± 1.7 years (ranged 2.5–10 years). Development of psychosis was determined by the CAARMS or the SIPS criteria, as described in our previous study (22). We recruited sex- and age-matched HCs from the community, hospital staff, and students at each site.

All subjects were physically healthy at the time of MRI scanning. Exclusion criteria were detailed in our previous study (22). All subjects provided written informed consent. If the participants were minors, written informed consent was provided by their parents. This study was approved by the Committee on Medical Ethics at each site.

### MRI Data Acquisition

The MRI scanners and data acquisition parameters used at each site were detailed in the Supplemental Material. All three sites used scanners with 1.5-tesla field strength.

### FreeSurfer-Initialized Labeled Cortical Distance Mapping (FSLCDM)

Images were initially preprocessed with FreeSurfer (version 5.3) (23). Each preprocessed image was carefully inspected, and any errors were manually corrected. FreeSurfer extracts the surfaces of gray and white matter, and automatically segments 68 cortical regions of interest (ROIs) using the Desikan–Killiany Atlas (24), including the STG.

### Extraction of PT

**Figure 1:**
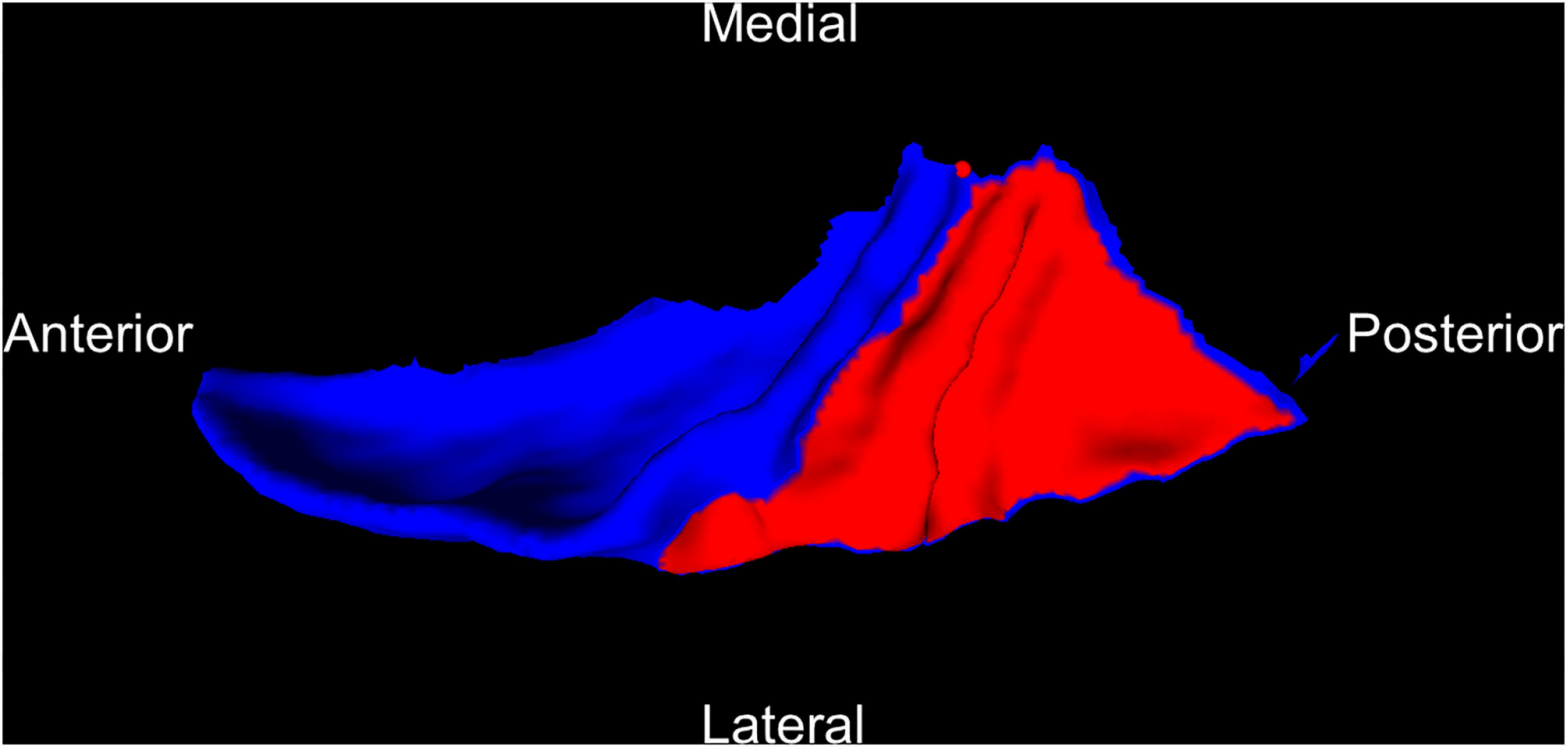
An example of PT (red) cut from the superior temporal gyrus (blue) on the left hemisphere. PT, planum temporale.

The PT was extracted from the Desikan–Killiany-defined STG following an established protocol (25). Boundaries of the PT were determined using a curvature-based surface tracking algorithm as follows: the boundary is defined by a path following the sulcus posterior to Heschl's gyrus from the medial to lateral extent, continues along a gyrus to the posterior ramus, and finally connects back to the starting point near the retro-insular (medial) end of Heschl's gyrus following a geodesic path. In cases with multiple Heschl's gyri or gyri that bifurcate at the lateral end, the more anterior gyrus is chosen as the PT boundary. Figure 1 shows an example of an extracted PT on the left hemisphere.

### LCDM

The cropped MRI was segmented into white matter (WM), gray matter (GM), and cerebrospinal fluid (CSF) using a mixture model averaging method (26, 27). The previously-developed method for generating LCDMs (28–30) was applied. To generate a distance map for the GM, the distance between each GM voxel and the closest GM/WM surface vertex was calculated at a 1 × 1 × 1 mm resolution. GM voxels associated with a vertex in the PT surface were labeled as PT. Voxels in the range of −2 to 8 mm were used for the analysis. The result of LCDM is a probability distribution function of the GM distance from the PT GM/WM surface. Three PT measures, i.e., cortical thickness, GM volume and surface area, were estimated in this way. Cortical thickness was determined using the distance at the 95th percentile of the distance distribution. Due to outlier voxels at distances >6 mm, the volume of voxels with distance less than or equal to the 95th percentile was taken as the volume of the PT. The area of the gray/white matter boundary surface was calculated from the triangulated surface.

### Neurocognitive Assessment

In 36 ARMS subjects, neurocognitive performance was evaluated using the Japanese version of the brief assessment of cognition in schizophrenia (BACS-J) (31) by trained psychiatrists or psychologists at baseline. The BACS-J includes brief assessments of verbal memory (list learning), working memory (digit sequencing task), motor function (token motor task), verbal fluency (category/letter fluency), attention and processing speed (symbol coding), and executive function (the Tower of London test).

### Statistical Analysis

One-way analysis of variance (ANOVA), independent two-sample *t*-tests, or a chi-squared test were used to compare clinical measures across the diagnostic groups. PT measures (i.e., cortical thickness, volume, and surface area) were compared among the controls, ARMS-NP and ARMS-P groups using repeated measures analysis of covariance (ANCOVA), with diagnosis as the between-subject factor, hemisphere as the within-subject factor, and age, sex, intracranial volume (ICV), daily antipsychotic dosage, and scanning site as nuisance covariates. Repeated measures were applied to evaluate laterality (i.e., left vs. right). For the *post-hoc* pairwise comparison, we used Bonferroni's correction. The associations between PT measures and neurocognitive functions (i.e., BACS scores) in ARMS subjects were evaluated by calculating partial correlation coefficients adjusted for age, sex, ICV, antipsychotic use, and scanning site. Bonferroni's correction was again adopted for these correlational analyses. The significance level was set at *p* < 0.05 (two-tailed).

## Results

### Clinical Characteristics

Table 1 summarizes the clinical characteristics of the diagnostic groups. Eighteen of the 73 ARMS subjects developed frank psychosis (i.e., ARMS-P subjects) after MRI scanning. Based on the Diagnostic and Statistical Manual of Mental Disorders fourth edition (DSM-IV) (20) criteria, the diagnoses of ARMS-P subjects comprised twelve schizophrenia cases, one delusional disorder case, one schizophreniform disorder case, and four cases with psychotic disorder NOS. Thirty of the 73 ARMS subjects (41%) were taking antipsychotics at baseline. Self-reported educational attainment was significantly higher in HC than in the ARMS-NP (*p* < 0.001) and ARMS-P (*p* < 0.001) groups. The BACS subscores did not differ between ARMS-NP and ARMS-P groups.

**Table 1:** Demographic and clinical characteristics.

**Variables**	**Group**				
	**HC**	**ARMS-NP**	**ARMS-P**	**Statistics**	***p***
Total number of subjects	74	55	18		
Site					
Toyama	52	11	5		
Toho	5	19	4		
Tohoku	17	25	9		
Age (mean ± SD)	22.6 ± 4.3	22.3 ± 6.5	20.1 ± 4.3	*F* = 1.7	0.19
Sex (male/female)	37/37	21/34	5/13	*X^2^* = 3.7	0.16
Handedness^*^ (right/both/left)	58/0/0	34/8/2	10/2/2		
Education years^**^ (mean ± SD)	14.8 ±1.9	12.2 ± 2.6	12.3 ± 2.3	*F* = 23.8	<0.001
Parental education years^***^ (mean ± SD)	12.9 ± 2.2	13.5 ± 2.0	13.4 ± 1.6	*F* = 0.86	0.43
Weeks between scanning and onset of psychosis (mean ± SD)			40.1 ± 32.6		
Antipsychotics dose (mean mg ± SD, chlorpromazine equivalent)		145 ± 102	196 ± 130	*t* = 1.1	0.26
BACS-J subscores^****^					
Verbal memory (mean ± SD)		46.4 ± 10.1	50.7 ± 12.6	*t* = 1.0	0.32
Digit sequencing task (mean ± SD)		18.3 ± 5.0	21.0 ± 5.4	*t* = 1.4	0.18
Token motor task (mean ± SD)		70.9 ± 14.1	70.0 ± 8.1	*t* = 0.18	0.86
Category/letter fluency (mean ± SD)		40.2 ± 12.8	44.3 ± 10.2	*t* = 0.87	0.39
Symbol coding (mean ± SD)		63.3 ± 12.9	64.9 ± 19.2	*t* = 0.28	0.78
Tower of London (mean ± SD)		17.8 ± 2.5	18.1 ± 2.4	*t* = 0.28	0.73

* *Data missing for 30 subjects*.

** *Data missing for 7 subjects*.

*** *Data missing for 38 subjects*.

**** *36 ARMS subjects (27 ARMS-NP and 9 ARMS-P subjects) underwent BACS-J*.

*ARMS, at risk mental state; BACS-J, brief assessment of cognition in schizophrenia Japanese version; HC, healthy controls; NP, did not develop psychosis; P, developed psychosis; SD, standard deviation*.

### PT Measures

Repeated measures ANCOVA adjusted for age, sex, ICV, daily antipsychotic dosage and scanning site demonstrated significant main effects of side for volume (*F*_(__2,144)_ = 25.46, *p* < 0.001) and surface area (*F*_(__2,144)_ = 26.47, *p* < 0.001) of PT. *Post-hoc* testing showed that volume and surface area were larger on the left side (*p* < 0.001) than on the right in all diagnostic groups. Although there was a significant main effect of diagnosis for surface area (*F*_(2,138)_ = 4.48, *p* = 0.013), *post-hoc* test did not reach a statistically significant level. No other significant diagnostic effects were found. There were no significant side × diagnosis interactions (Table 2). There were no significant correlations between BACS subscores and PT measures in these 36 ARMS subjects.

**Table 2:** Comparisons of PT measures among healthy controls, non-converters, and converters.

	**HC (*****n*** **=** **74)**		**ARMS-NP (*****n*** **=** **55)**		**ARMS-P (*****n*** **=** **18)**		**ANCOVA**^****a****^						***Post hoc***
							**Diagnosis**		**Side**		**Side** **×** **Diagnosis**		
**Measures**	**Mean**	**SD**	**Mean**	**SD**	**Mean**	**SD**	***F***	***P***	***F***	***p***	***F***	***p***	
Left PT thickness (mm)	3.10	0.79	3.01	0.77	3.09	0.76	0.164	0.849	0.364	0.547	0.064	0.938	
Right PT thickness (mm)	3.08	0.84	2.89	0.89	3.03	0.78							
Left PT volume (mm^3^)	2529	1025	2437	864	2520	936	0.851	0.429	**25.462**	** <0.001**	0.120	0.887	Left > Right
Right PT volume (mm^3^)	1859	770	1741	882	2009	913							
Left PT area (mm^2^)	850	236	880	212	939	316	4.48	0.013*	**26.469**	** <0.001**	0.184	0.832	Left > Right
Right PT area (mm^2^)	658	223	703	336	755	277							*NS

a *Age, sex, intracranial volume, daily antipsychotic dosage and scanning site were entered as covariates*.

ARMS, at risk mental state; HC, healthy controls; NP, did not develop psychosis; P, developed psychosis; PT, planum temporale; SD, standard deviation. The Bold values indicate significant main effects. *Did not reach a statistically significant level.

## Discussion

We demonstrated significant leftward asymmetries of PT volume and surface area in both ARMS-P and ARMS-NP subjects. In addition, we did not find PT structural changes predating the onset of psychosis in the ARMS-P group. These results were consistent with previous studies (15, 32) in which PT structural changes in ARMS-P subjects compared to ARMS-NP or healthy subjects were not found at baseline. The mean duration between MRI scanning and the onset of psychosis was 40 weeks, which was comparable to those of the same previous studies (33 and 28 weeks) (15, 32). Taken together, PT structural changes may occur at a closer time point (i.e., >30–40 weeks) before the onset of frank psychosis, or simultaneously with or immediately after the initial appearance of psychotic symptoms.

Other studies have demonstrated brain structure changes preceding the onset of psychosis, such as volume reduction or cortical thinning of the anterior cingulate cortex (22, 33, 34) and parahippocampal gyrus (35). These changes may reflect pre-existing vulnerability to psychosis, and may be useful as prognostic markers. In contrast, previous (15, 32) and current findings suggest that progressive PT structural changes, which have been reported in first-episode schizophrenia (8, 9), are closely associated with the first manifestation of psychotic symptoms (i.e., onset-related).

Although PT is normally an asymmetric (left > right) structure (4), this laterality is reportedly moderated, extinguished, or even reversed in patients with schizophrenia (6). Previous longitudinal studies suggest that the magnitude of progressive gray matter loss of PT is more prominent on the left side among first-episode psychosis and ARMS-P subjects (8, 9, 15). For instance, among ARMS-P subjects, an annual gray matter reduction of −5.2% on the left- and −3.9% on the right-side PT was reported (15). Therefore, the absence or reversal of leftward asymmetry of PT in established schizophrenia patients may have formed in the early stage of their psychosis.

Oertel et al. (7) demonstrated an intermediate reduction of the leftward asymmetry of the PT volume in first-degree relatives of schizophrenia patients (i.e., healthy subjects > relatives > probands) (7), while our data and previous studies (15, 32) did not detect such reduced laterality of PT measures in clinical high risk subjects. The mechanism of the emergence of reduced leftward asymmetry of PT structure might be different between clinical and genetical high risk individuals.

When conducting a study using ARMS patients, it can be challenging to obtain sufficient statistical power at a single site. We enrolled participants at three sites, and were able to obtain relatively a large sample size. Thus, the multi-site design is a strength of the present study, in addition to the employment of the novel LCDM imaging method.

Although previous studies demonstrated cognitive deficits in some domains (e.g., attention and working memory) in ARMS-P groups prior to the onset of psychosis (36, 37), we failed to replicate these results. A recent study by Jung et al. (38) found a positive correlation between verbal intelligence and PT volume in first-episode schizophrenia spectrum psychosis patients. We did not observe associations between PT measures and verbal performance in the ARMS group. The small number (*n* = 36) of ARMS subjects who underwent BACS and the difference in phase of psychosis may have influenced these inconsistent results. Because antipsychotics can affect both brain morphology (39) and cognitive function (40), it is also possible that the higher rate of antipsychotic use in our ARMS cohort biased the findings.

Our previous studies (15, 32) employed subjects recruited at the University of Melbourne, therefore the subjects of our previous and current studies do not overlap. Although current and previous studies are similar with regard to the subjects' age distribution, ARMS subjects in previous studies (15, 32) were antipsychotic-naïve at baseline while some of ARMS subjects in current study were taking antipsychotics. Nonetheless, the results of PT measures at baseline were similar among current and previous studies.

We should note some limitations. First, the MRI scanners, acquisition parameters, and the proportion of controls to ARMS subjects differed among the sites, and these differences may have confounded the results, although we treated site as a nuisance covariate in each statistical model and there were no diagnosis × site or hemisphere × diagnosis × site interactions. Second, the combined use of two criteria for ARMS (i.e., SIPS/SOPS or CAARMS) may have affected the results, although these two measures largely overlapped. Finally, the effects of antipsychotic medications on brain morphology could not be fully excluded (39), although we considered usage of antipsychotics in the statistical models.

In conclusion, our results did not support morphological changes of PT predating the onset of psychosis. Due to the cross-sectional design of this study, we were unable to examine whether PT measures change in accordance with the manifestation of psychotic symptoms. Longitudinal analyses with repeated scans are warranted in future studies.

## Data Availability Statement

The datasets generated during the current study will not be available for public use, since we do not have permission to share the data. Requests to access the datasets should be directed to the corresponding author, Yoichiro Takayanagi, takayanagi-matsu@umin.net.

## Ethics Statement

The studies involving human participants were reviewed and approved by the Institutional Review Board of the Toho University School of Medicine, Ethics Committee, University of Toyama, the Ethics Committee of Tohoku University Graduate School of Medicine. Written informed consent to participate in this study was provided by the participants' legal guardian/next of kin.

## Author Contributions

MS and YT designed the study and wrote the protocol. TT, NK, AS, NO, MKa, SN, KM, and MM recruited subjects and were involved in clinical and diagnostic assessments. AF, MKi, and MN managed the MRI and clinical data. YT, SK, DS, and JR did the imaging processing. KN provided technical support for MRI scanning and data processing. YT performed statistical analyses and wrote the first draft of the manuscript. SK, TT, JR, and MS contributed to editing the manuscript. All authors have approved the final manuscript.

## Conflict of Interest

The authors declare that the research was conducted in the absence of any commercial or financial relationships that could be construed as a potential conflict of interest.
