# Supplementary material for: Structural MRI Study of the Planum Temporale in Individuals With an At-Risk Mental State Using Labeled Cortical Distance Mapping
Source: Front Psychiatry. 2020 Nov 24;11:593952. doi: 10.3389/fpsyt.2020.593952 (PMC7732500; doi:10.3389/fpsyt.2020.593952)
Supplement: Supplementary file 1 [file Data_Sheet_1.doc]

**MRI scanners and image acquisition parameters**

University of Toyama

A 1.5-T scanner (Magnetom Vision, Siemens Medical System, Inc., Erlangen, Germany) was used with three-dimensional gradient-echo sequence FLASH (fast low-angle shots) yielding 160-180 contiguous T1-weighted slices of 1.0-mm thickness in the sagittal plane. The imaging parameters were as follows: repetition time = 24 ms; echo time = 5 ms; flip angle = 40°; field of view = 256 mm; and matrix size = 256 × 256 pixels. The voxel size was 1.0 × 1.0 × 1.0 mm.

Toho University

Participants underwent magnetic MRI scans using a 1.5-T scanner (EXCELART Vantage, XGV 1.5 T; Toshiba Medical Systems, Tokyo, Japan) yielding 160 contiguous T1-weighted slices of 1.0-mm thickness in the sagittal plane. The imaging parameters were: repetition time = 24.4 ms: echo time = 5.5 ms; flip angle = 35°; field of view = 250 mm; matrix size = 256 × 256 pixels. The voxel size was 0.98 × 0.98 × 1.0 mm.

Tohoku University

A 1.5-T scanner (Achieva, Phillips Medical Systems, Best, Netherlands) was used for MRI scanning with three-dimensional fast field echo sequencing yielding 200 contiguous T1-weighted slices of 1.0-mm thickness in the sagittal plane. The imaging parameters were as follows: repetition time = 30 ms, echo time = 5 ms, flip angle = 30˚, field of view = 256 mm, matrix size = 256 × 256 pixels. The voxel size was 1.0 × 1.0 × 1.0 mm.
